# Efficacy and safety of Tengfu Jiangya tablet combined with valsartan/amlodipine in the treatment of stage 2 hypertension: study protocol for a randomized controlled trial

**DOI:** 10.1186/s13063-022-06089-z

**Published:** 2022-02-22

**Authors:** Yu Wang, Zhen Hua, Wenjing Chen, Yushuo Zhu, Yunlun Li

**Affiliations:** 1 Affiliated Hospital of Shandong University of Traditional Chinese Medicine, Jinan, 250011 Shandong China; 2 Shandong University of Traditional Chinese Medicine, Jinan, 250355 Shandong China

**Keywords:** Stage 2 hypertension, Tengfu Jiangya tablet, Valsartan/amlodipine, Randomized controlled trial

## Abstract

**Background:**

The prevalence rate of hypertension in the Chinese population is on the rise, and the control rate of hypertension is low. International guidelines, including the 2018 Chinese Guidelines for the Management of Hypertension, recommend optimized drug selection and combination therapy for patients with stage 2 hypertension and blood pressure ≥ 160/100 mmHg, including valsartan/amlodipine (Val/Aml). The traditional Chinese medicine (TCM) compound Tengfu Jiangya tablet (TJT; No. Z20110021, Shandong Provincial Food and Drug Administration) is prepared in the medical institution of Affiliated Hospital of Shandong University of Traditional Chinese Medicine. It is an effective compound preparation of TCM for the treatment of hypertension in the national clinical research base of TCM. The aim of this study was to evaluate the efficacy and safety of TJT combined with Val/Aml in the treatment of stage 2 hypertension with hyperactivity of liver yang.

**Methods:**

This randomized double-blind, placebo-controlled, multicenter trial will be conducted with a total of 288 participants with stage 2 hypertension at seven clinical trial centers. The stratified random method will be used, and the subcenter will be taken as the stratification factor. Eligible patients will be randomly assigned (1:1) into groups receiving either TJT or placebo three times daily for 28 days, both combined with Val/Aml 80/5 mg. The primary efficacy endpoint is the reduction in the mean sitting systolic blood pressure (msSBP) and the mean sitting diastolic blood pressure (msDBP) from baseline to week 4. Adverse events and laboratory test results will be monitored throughout the trial.

**Discussion:**

This is the first placebo-controlled randomized trial conducted to evaluate the efficacy and safety of a Chinese herbal extract combined with Val/Aml in patients with stage 2 hypertension. Our study may help to provide evidence-based recommendations of a complementary preventive measure for stage 2 hypertension.

**Trial registration:**

Chinese Clinical Trial Registry ChiCTR2000030611. Registered on 8 March 2020

**Supplementary Information:**

The online version contains supplementary material available at 10.1186/s13063-022-06089-z.

## Administrative information

*Trials* guidance: please include this text in your protocol just above the administrative information table:

Note: the numbers in curly brackets in this protocol refer to SPIRIT checklist item numbers. The order of the items has been modified to group similar items (see http://www.equator-network.org/reporting-guidelines/spirit-2013-statement-defining-standard-protocol-items-for-clinical-trials/).

**Table:** 

Title {1}	Efficacy and safety of Tengfu Jiangya tablet combined with valsartan/amlodipine in the treatment of stage 2 hypertension: study protocol for a randomized controlled trial
Trial registration {2a and 2b}	Chinese Clinical Trial Registry, ChiCTR2000030611. Registered on 8 March 2020.
Protocol version {3}	Dated August 8, 2020. Protocol version 2.0.
Funding {4}	This research has received funding from the Shandong Clinical Medical Research Center for Cardiovascular and Cerebrovascular Diseases of Traditional Chinese Medicine, National Natural Science Foundation of China(Grant No.81774173 and Grant No.81774242), Shandong Province Health Science and Technology Development Plan of 2020 (Grant No.202003011161), and Shandong Province Traditional Chinese Medicine Science and Technology Development Program (Grant No.2015-100). The funder had no role in the design of the study; in the collection, analyses, or interpretation of the data; in the writing of the article; or in the decision to publish the results.
Author details {5a}	Yu Wang, Affiliated Hospital of Shandong University of Traditional Chinese Medicine, Jinan 250011, Shandong, China. E-mail: 623393949@qq.com Zhen Hua, Affiliated Hospital of Shandong University of Traditional Chinese Medicine, Jinan 250011, Shandong, China. E-mail: huazhen0326@163.com Wenjing Chen, Affiliated Hospital of Shandong University of Traditional Chinese Medicine, Jinan 250011, Shandong, China. E-mail: wenjing198412@163.com Yushuo Zhu, Affiliated Hospital of Shandong University of Traditional Chinese Medicine, Jinan 250011, Shandong, China. E-mail: 957712401@qq.com Yunlun Li, ^1^Affiliated Hospital of Shandong University of Traditional Chinese Medicine, Jinan 250011, Shandong, China, ^2^Shandong University of Traditional Chinese Medicine, Jinan 250355, Shandong, China. E-mail: yunlun.lee@hotmail.com Yu Wang and Zhen Hua are co-first authors of this manuscript.
Name and contact information for the trial sponsor {5b}	Yunlun Li, ^1^Affiliated Hospital of Shandong University of Traditional Chinese Medicine, Jinan 250011, Shandong, China, ^2^Shandong University of Traditional Chinese Medicine, Jinan 250355, Shandong, China. E-mail: yunlun.lee@hotmail.com
Role of sponsor {5c}	YL carried out the trial design and financial supervision.

## Introduction

### Background and rationale {6a}

Hypertension is a common, chronic, noncommunicable disease caused by the interaction of many factors; it is also the most important and controllable risk factor for cardiovascular disease. Hypertension causes a heavy burden of disease [1]. According to global data, from 1975 to 2015, the number of people suffering from hypertension in the world increased from 594 million to 1.1 billion. There are 226 million people with hypertension living in China; the proportion of stage 2 hypertension is as high as 11.0% [2]. A prospective cohort study (China Kadoorie Biobank Study) showed that the diagnosis rate, treatment rate, and control rate of hypertension in Chinese adults are significantly lower than those in the Western population, and the rate of noncompliance and mortality are higher [3]. At present, improving the attainment rate of blood pressure (BP) in hypertensive patients is the most important task in the health management of cardiovascular disease; BP control of patients with stage 2 hypertension is still challenging [4]. As shown by VALUE, FEVER, and other studies, effective BP reduction within 4 weeks provides clear cardiovascular benefits, significantly reducing all-cause death, while reaching the target at 3 and 6 months does not reduce all-cause death [5, 6]. Therefore, international guidelines recommend that patients with stage 2 hypertension should start combined antihypertensive therapy and implement the antihypertensive treatment strategy based on single-compound preparation (SPC) to reduce the burden of tablets and improve compliance to further strengthen the control of BP; they also recommend a positive evaluation (a hypotension evaluation period of 2–4 weeks) to achieve the standard BP early, ideally within 4 weeks [7, 8].

Clinical and basic research has demonstrated that changes in vascular function and structure occur early in hypertensive diseases, including vascular endothelial cell damage, vascular endothelial dysfunction, abnormal vasoconstriction, and vascular remodeling [9, 10]. In addition to the development of hypertension, progressive damage and deterioration in vascular endothelial function and structure further facilitate the development of hypertension and increase the risk of cardiovascular events, including myocardial infarction, heart failure, and stroke. Thus, protecting vascular endothelial cells and correcting endothelial dysfunction have emerged as promising therapeutic targets for achieving standard BP early and treating hypertension [11].

Traditional Chinese medicine (TCM) is an important part of complementary and alternative medicine. Studies have shown that Chinese herbal medicine can play a role in protecting vascular endothelial cells and correcting endothelial dysfunction through multiple pathways and targets [12]. TCMs in the treatment of hypertension can be combined with antihypertensive drugs. This plays a positive role in reducing the dosage of antihypertensive drugs, enhancing the curative effect and BP stability [13]. In addition, in different periods of the occurrence and development of hypertension and different syndrome types, approximately 87.23% of patients had liver-yang hyperactivity syndrome [14].

Tengfu Jiangya tablets (TJTs) are successfully prepared by modern preparation technology to extract the effective antihypertensive components of *Uncaria rhynchophylla* and *Semen Raphani*. The main indication of TJT is hyperactivity syndrome of liver yang in hypertension. In a previous study [15–18], based on the metabonomics and proteomics techniques, we found that TJT may reduce BP by improving the production of NO and might exhibit additional cardiovascular protective effects by improving vascular endothelial inflammation and vascular remodeling. The molecular mechanism is mainly related to the kallikrein-kinin pathway, lipid metabolism pathway, and PPARγ signal transduction pathway. In an ongoing multicenter, randomized, double-blind clinical trial (ChiCTR-IIR-17011940) of TJT for the treatment of liver-yang hyperactivity in stage 1 hypertension, a preliminary analysis of the results showed that the antihypertensive effect of TJT was better than that of placebo. In addition, other small-sample randomized controlled trials also showed that TJT improved the clinical symptoms of hypertensive patients with hyperactivity of the liver and lower BP [19]. However, there is still a lack of high-quality evidence in large-sample, blinded, randomized, placebo-controlled clinical trials for stage 2 hypertension to reasonably evaluate whether TJT as an adjuvant treatment of ARB/CCB and SPC can further promote the early achievement of BP and improve clinical symptoms and patient compliance in patients with stage 2 hypertension.

Therefore, this study will involve a multicenter, double-blind, randomized, placebo-controlled method to evaluate the efficacy and safety of TJT-assisted Val/Aml in the treatment of stage 2 hypertension, providing a practical basis for the prevention and treatment of hypertension.

A completed SPIRIT checklist is available as a supplement (Additional file 1).

### Objectives {7}

The aims of our study were to evaluate the clinical efficacy and safety of TJT combined with Val/Aml SPC in the treatment of essential hypertension (stage 2) with hyperactivity of liver yang and to observe whether Val/Aml combined with TJT can further promote the early achievement of BP and improve clinical symptoms and patient compliance in patients with stage 2 hypertension compared with Val/Aml combined with placebo.

### Trial design {8}

This multicenter, double-blind, randomized, placebo-controlled, superiority trial was reviewed and approved by the Ethics Committee of the Affiliated Hospital of Shandong University of Traditional Chinese Medicine [approval registration number (2019) 伦审 (064) 号-KY] and registered in the Chinese Clinical Trial Registry (ChiCTR2000030611, version 2.0, dated 8 August 2020). The Chinese Clinical Trial Registry is a primary registry of the WHO ICTRP network and includes all items from the WHO Trial Registration dataset that are available at the link (http://www.chictr.org.cn/listbycreater.aspx). The trial will be conducted according to the principles of the Declaration of Helsinki and Good Clinical Practice Guidelines. In addition, we will comply with the Consolidated Standards of Reporting Trials (CONSORT) Extension of Chinese Herbal Medicine Formulas 2017 [20] when reporting the results. This study planned to recruit 288 subjects who will be randomly assigned in a 1:1 ratio.

**Fig. 1:**
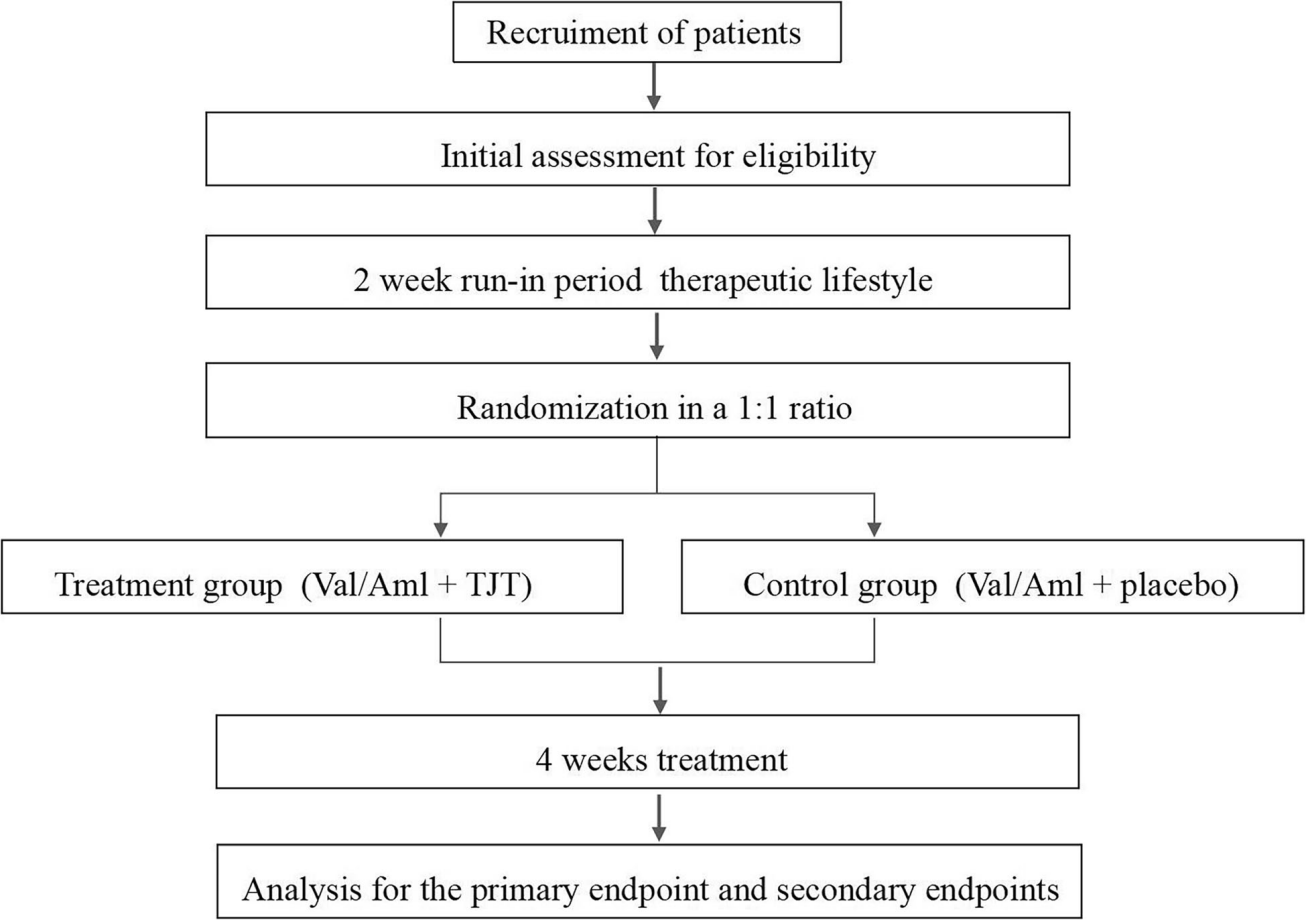
Flowchart of this study

An overview of the study procedures is shown in Fig. 1.

## Methods: participants, interventions, and outcomes

### Study setting {9}

The trial will be conducted in seven centers in Shandong Province, China, and all these hospitals and their ethics approvals are shown in Additional file 2. A total of 288 participants will be recruited. All the patients will provide written informed consent before participating in this study; recruited patients will be randomly divided into a treatment group (treated with Val/Aml and TJT) or a control group (treated with Val/Aml and placebo) in a 1:1 ratio. After the consent of participants is obtained, a trial will be conducted, including a run-in period of 2 weeks and a treatment period of 4 weeks. The onsite follow-up test of the research center will be conducted at 2 and 4 weeks after random grouping. In addition, telephone follow-up will be conducted on day 3 and week 3.

### Eligibility criteria {10}

#### Diagnostic criteria

The diagnostic criteria for primary stage 2 hypertension are based on the 2018 Chinese Guidelines for the Management of Hypertension (2018 revised edition) [21]. The diagnostic criteria for hyperactivity of liver yang in hypertension are based on the Hypertension TCM syndrome Diagnosis Scale developed by the National Hypertension TCM Clinical Research Base [22]. (Additional file 3)

#### Inclusion criteria

The following are the inclusion criteria:

1. Age ≥ 18 years and ≤ 65 years, male or female
2. At the same time, conforming to the diagnostic criteria for stage 2 hypertension [21] and the TCM syndrome differentiation criteria for liver-yang hyperactivity syndrome [22]
3. For patients with primary stage 2 hypertension diagnosed according to established guidelines [21], BP that satisfies any one of the following three criteria:
  - Patients who have SBP 160–179 mmHg and/or diastolic BP (DBP) 100–109 mmHg, without prior use of any antihypertensive drugs
  - Patients who have been clearly diagnosed with essential hypertension and whose BP is not up to standard after 3 months of improved lifestyle or other forms of nondrug therapy are maintained at 160–179/100–109 mmHg
  - Patients who have been clearly diagnosed with essential hypertension and whose BP is still not up to the standard BP after 3 months of improved lifestyle or other forms of nondrug therapy and oral antihypertensive drugs maintain at 160–179/100–109 mmHg
4. Patients must have had a definite diagnosis of hypertension for more than 3 months
5. Patients will be informed about the trial and should voluntarily sign a consent form.

#### Exclusion criteria

The following are the exclusion criteria:

1. Secondary hypertension
2. Participation in other clinical trials in the previous 3 months.
3. The following diseases or organ damage within 6 months prior to screening: cardiovascular disease (unstable angina pectoris, heart failure, valvular disease, myocardial infarction), cerebrovascular disease (stroke, transient ischemic attack), hypertensive encephalopathy, retinopathy (with or without apparent papillary edema), aortic aneurysm, or aortic dissecting aneurysm artery occlusive disease symptoms
4. Pregnancy, preparing for pregnancy, and lactating
5. Allergies to multiple drugs or having an allergic constitution
6. Mental illness, alcoholism, and/or psychoactive substance abuse and dependence
7. Severe liver disease or aspartate aminotransferase (AST) or alanine aminotransferase (ALT) ≥ normal upper limit. Severe kidney disease or creatinine level ≥ 2.0 mg/dL. Uncontrolled diabetes with HbA1c ≥ 9% or fasting glucose ≥ 160 mg/dL
8. Having received Val/Aml (80/5 mg) and other ARB/CCB type combination drugs and still having level 2 or above BP

#### Suspension criteria

The following are the suspension criteria:

1. Poor compliance of patients.
2. The mean sitting SBP (msSBP) ≥ 200 mmHg and/or the mean sitting DBP (msDBP) ≥ 120 mmHg will result in exclusion at any time during the study period.
3. Adverse events (AEs), complications, or physiological changes that are fatal.
4. Use of prohibited drugs or treatments that may affect the analysis of results during the trial.
5. Voluntary withdrawal.
6. Incomplete data.
7. Withdrawal due to various reasons, such as failure to participate in the follow-up.

### Who will take informed consent? {26a}

Informed consent documents will be obtained by the principal investigator at each subcenter. The participating consultant will explain the details of the informed consent document to the potential participants at each subcenter, and the principal investigators will obtain written permission from patients willing to participate in the trial.

### Additional consent provisions for collection and use of participant data and biological specimens {26b}

The subjects will be asked whether they agree to the use of their information. It should be noted that no biological samples will be collected in our study.

### Interventions

#### Explanation for the choice of comparators {6b}

After a 2-week run-in period, patients with stage 2 hypertension will be randomly divided into a treatment group and a control group, and they will receive Val/Aml plus TJT or Val/Aml plus TJT placebo for 4 weeks based on their lifestyle changes.

#### Intervention description {11a}

Participants in the treatment group will receive Val/Aml 80/5 mg once daily, combined with TJT tablets 1200 mg twice daily for 4 weeks. Participants in the control group will receive Val/Aml 80/5 mg once daily, combined with TJT placebo tablets 1200 mg twice daily for 4 weeks. TJT (production batch number 200603) and TJT placebo (production batch number 200604) were produced and packaged by the preparation center of the Affiliated Hospital of Shandong University of Traditional Chinese Medicine. After testing, the quality of the drug satisfied the requirements of TCM standards issued and implemented by the State Food and Drug Administration (SFDA), and the quality standards and testing methods of the placebo were consistent with those of the experimental drug. TJT has a composition of 300 mg/tablet; each tablet contains 230 mg *Uncaria angustifolia* extract and 70 mg *Semen Raphani* extract. The TJT placebo has a composition of 300 mg/tablet, each containing 136 mg yellow dextrin, 136 mg white dextrin, and 28 mg caramel. Val/Aml was purchased from Beijing Novartis Pharmaceutical Co., Ltd. (Beijing, China, Unified social credit code 9111000060001684X1).

#### Routine care

Routine care for participants in both groups during the trial will follow the Chinese Society of Hypertension’s 2018 Revision Guidelines for Prevention and Treatment of Patients with Cardiovascular and Cerebrovascular Disease, including reducing sodium salt intake, controlling body mass, quiting smoking, quiting drinking, reducing mental stress, and maintaining psychological balance and middle-intensity exercise.

#### Criteria for discontinuing or modifying allocated interventions {11b}

Discontinuation criteria are withdrawal of consent, subsequent occurrence of a suspension criteria criterion (e.g., use of prohibited drugs), lack of compliance, and medical problems for stopping the intervention.

#### Monitoring compliance

During each follow-up, the researcher will count the number of medicines distributed and recovered; record the number of medicines received, used, and returned by the subjects to measure the compliance; and record it in the CRF in time. Subjects with compliance rates lower than 80% will be considered to have low compliance.

#### Strategies to improve adherence to interventions {11c}

The researchers will improve patient compliance through regular follow-up, BP checks, and medication diary CARDS.

#### Relevant concomitant care permitted or prohibited during the trial {11d}

During the trial, patients will be requested to not use any additional herbal decoction or proprietary Chinese medicine, acupuncture, or other methods to treat hypertension.

#### Provisions for posttrial care {30}

According to the clinical use of *Uncaria rhynchophylla*, *Semen Raphani*, and other traditional Chinese medicine decoction pieces and Val/Aml, we do not expect serious adverse events due to our intervention.

### Outcomes {12}

#### Primary outcomes

The main functional variables in the study include the changes in msSBP and msDBP from baseline to week 4 (end point).

#### Secondary outcomes

The following are the secondary outcomes:

1. BP control rate at week 4 (defined as msSBP/msDBP < 140/90 mmHg).
2. To evaluate the improvement of symptoms, the TCM syndrome scoring scale [22] will be used for evaluation (Additional file 3). To determine the degree of symptom improvement, we will refer to the Guiding Principles for Clinical Research on New Chinese Medicines [23]. Efficacy index = [(points before treatment − points after treatment)/points before treatment] × 100%. Significant effect: efficacy index ≥ 70%. Effectiveness: efficacy index ≥ 30%. Invalid: efficacy index < 30%.
3. Other results include blood lipids, blood glucose, urinary microalbumin, uric acid, homocysteine, and hypersensitive C-reactive protein. It should be noted that no biological samples will be collected in our study.
4. In addition, patient compliance will be assessed by counting the number of unused tablets returned at the scheduled visit. Table 1 shows the timing for all outcome measures in the study.

**Table 1:** Outcome measures

Domain	Measurement	Time (days)
**Primary outcomes**		
msSBP/msDBP	Three consecutive sitting BP measurements will be performed at 3-min intervals, and the average of three measurements will be used as a reference for each patient’s msSBP and msDBP.	0, 3, 14, 21, 28
**Secondary outcomes**		
BP control rate	Defined as msSBP/msDBP < 140/90 mmHg	28
TCM syndrome scoring	TCM syndrome scoring scale will be used for evaluating the improvement of symptoms (Additional file 3).	0,28
**Biochemical index**		
Blood lipids	On days 0 and 28 of the treatment cycle, the researchers will collect the morning fasting blood of each subject for biochemical detection.	0,28
Blood sugar		
Urine microalbumin		
Uric acid		
Homocysteine		
C-reactive protein		

#### BP measurement

We will use a proven medical electronic sphygmomanometer equipped with an upper arm. During the first visit, BP should be measured in both upper arms; the side with a higher reading will be selected as the upper arm for further study. Three consecutive sitting BP measurements will be performed at 3-min intervals, and the average of three measurements will be used as a reference for each patient’s clinical BP. While measuring BP, pulse rate will be measured.

#### Participant timeline {13}

The content and key points of data collection in the experiment are as follows:

1. Screening period (− 14 to 0 days): inclusion evaluation will be conducted in the first 14 days.
2. Intervention period (28 ± 2 days): telephone follow-up will be conducted on the 3rd day and 3rd week; field follow-up tests at the study center will be performed at weeks 2 and 4.

**Fig. 2:**
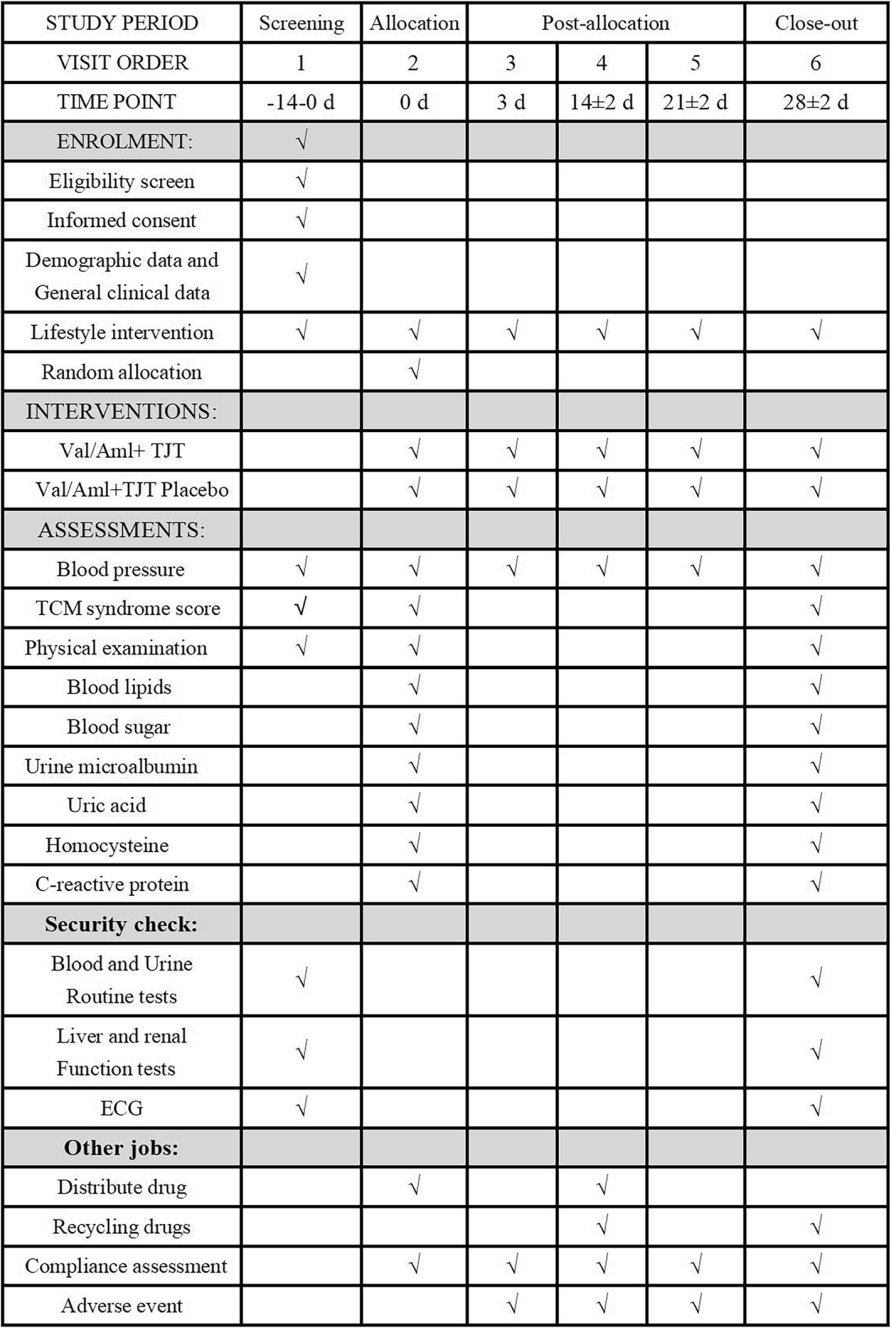
Schedule of enrollment, interventions, and assessments

Different items will be measured according to the point in time when the data are collected. The details are shown in Fig. 2.

#### Sample size {14}

The trial will check for superiority between the treatment group (treatment with Val/Aml and TJT) and the control group (treatment with Val/Aml and TJT placebo).

The calculation of sample size is based on the hypothesis of superiority and the method of comparing the two means. Assuming that the class I error rate is *α* = 0.05 and the class II error rate is *β* = 0.2, and based on a previous study [19], the test group DBP after treatment (*πc*) can be set as follows: DBP, 8.3 ± 5.53 mmHg, and the control group DBP after treatment (*πt*) can be set as follows: DBP, 6.3 ± 4.31 mmHg based on the effectiveness test sample size calculation formula:

$$ N=\left[2{\left({Z}_{1-\alpha }+{Z}_{1-\beta}\right)}^2{\delta}^2\right]/{\left({\pi}_T-{\pi}_c\right)}^2 $$

**δ**^2^ = (S1^2^ + S2^2^)/2; S1 and S2 are the standard deviations of the control group and treatment group, respectively; *α* = 0.05, *Z*_1−*α*_ = 1.96, *β* = 0.1, *Z*_1-*β*_ = 1.28, (*Z*_1 − *α*_ + *Z*_1 − *β*_)^2^ = 6.2. Then, each group needs a sample size of 120. The quality of the study will be strictly controlled during the trial. After considering the Good Clinical Practice (GCP) shedding rate (20%) and expanding the number of samples, we decided to recruit 288 patients in this trial, including 144 patients in the treatment group and 144 patients in the control group. The total number of cases in the two combinations will be 288: 144 in the treatment group and 144 in the control group.

#### Recruitment {15}

Researchers and attending physicians will select potential eligible patients based on the inclusion criteria. Patients will be informed in detail about the purpose of the study, procedures, possible side effects, and the benefits of the study, Val/Aml, and TJT. All patients who volunteer to participate are required to sign written informed consent prior to randomization (Additional file 4).

### Assignment of interventions: allocation

#### Sequence generation {16a}

The patients will be randomly divided into two groups by statisticians independent of the intervention and evaluation procedures in clinical trials. The ratio between the treatment group and control group is 1:1. Using the stratified grouping random method, the subcenter will be taken as the stratification factor.

#### Concealment mechanism {16b}

Each participant’s assignment code will be blinded and placed in a sealed opaque envelope. Each envelope will be opened by the researchers to assign the registered participants to a treatment group and control group. In each group, the placebo has identical appearance and smell as TJT.

#### Implementation {16c}

According to the number of patients undertaken by each research center, the statistical software (SAS, version 9.2) will be used to generate a random serial number consistent with the sample size and its corresponding group, i.e., the blind bottom, the random sequence number as the drug number of the study, and remaining blind until the end of the study.

### Assignment of interventions: blinding

#### Who will be blinded {17a}

The following research team members are blinded to the care of the subjects: the clinical investigators, the research staff, and attending physicians.

#### Procedure for unblinding if needed {17b}

The researcher can open the blinding code only when the subject has serious adverse events or other special or emergency events, which necessitates knowing the specific grouping of the subjects before treatment. Researchers should contact the ethics committee to report the reasons for unblinding and record the date of unblinding, treatment, and final results in the CRF form. The subjects should discontinue the study after unblinding. The main investigator will decide whether the research protocol needs to be revised according to safety data after unblinding.

### Data collection and management

#### Plans for assessment and collection of outcomes {18a}

After random allocation, subjects will be followed up for 4 weeks by the researchers and attending physicians at each subcenter. Onsite follow-up tests will be conducted at 2 and 4 weeks, and telephone follow-up will be conducted on day 3 and week 3. The data in CRF come from original documents, such as original medical records and physical and chemical examination reports, and shall be consistent with the original documents. The researchers are responsible for the timely, correct, legible, and complete recording of the data in the CRF and confirm recording by signature. All items in CRF shall be filled in without blank or missing items.

#### Plans to promote participant retention and complete follow-up {18b}

The following are the plans to promote participant retention and complete follow-up:

1. The researchers will explain the study protocol to the subjects to make them fully understand and cooperate with the test.
2. The researchers will follow up regularly to remind subjects to take medicine on time.
3. The subcenter is equipped with a special clinic for clinical research subjects to reduce their waiting time and create a good medical environment.
4. A diary card will be made, including medication records and precautions during the study.
5. Medical services: the researcher will provide health education to the subjects, including health education knowledge related to hypertension.

#### Data management {19}

To ensure the accuracy of the data, two data managers will independently perform double entry and proofreading. The data management center will be informed of any questions or unexpected situations in the CRF table. The inspector is responsible for monitoring whether the center is compliant with relevant regulations, GCPs, and test schemes to conduct this study. The inspector will cross-check the contents in CRF with the original documents to ensure the consistency of data in CRF with the original data. Data locking will be performed by the data manager at the end of the study. Researchers will not be able to modify the data later, and the Center for Evidence-Based Medicine will be responsible for verifying the data.

#### Confidentiality {27}

The researcher will keep the personal information of all registered subjects safely to protect confidentiality before, during, and after the test.

#### Plans for collection, laboratory evaluation, and storage of biological specimens for genetic or molecular analysis in this trial/future use {33}

No biological specimens are collected in this trial.

## Statistical methods

### Statistical methods for primary and secondary outcomes {20a}

#### Analysis population

The analysis population includes an intention-to-treat (ITT) analysis population and a per-protocol (PP) analysis population. The ITT analysis includes the subjects who express intention to receive treatment, sign informed consent, and are randomized. The PP analysis includes the subjects who complete the trial according to the study protocol.

#### Principles and methods of statistical analysis

All analyses will be conducted with SPSS version 26.0 (IBM SPSS Inc., Armonk, NY, USA) or SAS version 9.4 (SAS Institute, Inc., Cary, NC, USA). The data analysis, data entry, and data management of each research center will be conducted in the Evidence-based Medicine Research Center of Shandong University of Traditional Chinese Medicine. Bilateral tests will be used for all statistical tests, with *P* < 0.05 being considered statistically significant (unless otherwise specified). The 95% confidence interval will be calculated. The quantitative index will include the calculation of the mean, standard deviation, median, minimum, and maximum. The classification index is described by the number of cases and percentages of each category. The two study groups will be compared using an independent two-sample Student’s *t* test. For categorical variables, a *χ*^2^ test will be used when appropriate. A Wilcoxon paired signed-rank test will be used for within-group comparisons.

#### Interim analyses {21b}

The efficacy and safety of the Val/Aml combination have been demonstrated in large randomized controlled trials. In addition, the research treatment period was set to only 4 weeks. Therefore, we have no interim analyses or stopping guidelines.

### Methods for additional analyses (e.g., subgroup analyses) {20b}

We will consider whether to conduct subgroup analysis according to the subject’s age, BMI, and so on.

### Methods in analysis to handle protocol non-adherence and any statistical methods to handle missing data {20c}

Statistical analysis will be based on the ITT and PP principles. In ITT analyses, missing data will be supplemented by the last observation carried forward (LOCF) method.

#### Plans to give access to the full protocol, participant-level data, and statistical code {31c}

The datasets used during the current study are available from the corresponding author on reasonable request.

## Oversight and monitoring

### Composition of the coordinating center and trial steering committee {5d}

#### Trial steering committee

The trial steering committee (TSC) and coordination center are composed of the principal researcher, leading researcher of each subcenter, and experts from the Ethics Committee of the Affiliated Hospital of Shandong University of Traditional Chinese Medicine. The coordination center and TSC are responsible for the quality control of clinical trials, assisting and supervising the completion of the ethical review of each subcenter, reviewing the progress of the study, and providing unified training for the assistant investigators, including filling out the CRF form, subject follow-up, and adverse event reporting and management. In addition, they are in charge of the interpretation of the results and the publication of study reports. The study team and the main researchers will meet every 3 months to supervise, review, and discuss the study progress.

#### Subcenter

Each subcenter has a trained cardiologist for the recruitment of potential recruits and a study nurse for collecting ECG and blood samples. A research associate and graduate student research assistants will help with signing informed consent, filling out the CRF form, and general local organization.

#### Coinvestigators

The coinvestigators are responsible for supervising the progress of the trial and reviewing adherence to the study protocol. They report the research progress to TSC every 3 months.

#### Composition of the data monitoring committee, its role, and reporting structure {21a}

The data monitoring committee will consist of a senior cardiologist, one mid-career cardiologist, and the statistician of the study center. After the data are completely collected, digital data will be cross-checked by cardiologists using paper CRFs. Severe adverse events will be the major task for the data monitoring committee. Only the statistician will check the finalized combined dataset.

#### Adverse event reporting and harms {22}

Adverse events include any symptom, syndrome, or disease that is present in a subject during the clinical study and would affect the subject’s health, as well as clinically relevant conditions found during laboratory or other diagnostic procedures. Researchers must record the time, duration, measures taken, and severity of AE in the common reporting format (CRF) table within 24 h of AE occurrence and report to the Ethics Committee. The researchers will evaluate the correlation with the drug under study and decide whether to discontinue the observation based on the condition, and the cases of drug discontinuation due to adverse reactions will be unblinding and followed. According to the clinical use of *Uncaria rhynchophylla*, *Semen Raphani*, and other traditional Chinese medicine decoction pieces and Val/Aml, we do not expect serious adverse events due to our intervention.

#### Frequency and plans for auditing trial conduct {23}

Trial coordinator visits will audit the conduct of this trial every 3 months. The trial steering group and the independent ethics committee will meet regularly for review throughout the trial.

#### Plans for communicating important protocol amendments to relevant parties (e.g., trial participants, ethical committees) {25}

Important protocol modifications to the current study protocol will be submitted to the clinical research ethics committee, all trial participants, and the trial investigators.

#### Dissemination plans {31a}

Our study will be published in peer-reviewed journals to communicate the trial results to participants, health care professionals, the public, and other relevant groups. We will comply with the official authorship eligibility guidelines of all publications and do not intend to use professional writers.

## Discussion

Hypertension is an important risk factor for cardiovascular diseases; it is more difficult to control hypertension of grade II or above [1]. Therefore, better treatment methods are needed. Chinese herbal medicines for hypertension have attracted much attention, and an increasing number of studies are trying to combine Chinese herbal medicine with traditional antihypertensive drugs [24]. This is the motivation for considering TJT in this study.

Chinese herbal medicines contain a variety of natural active ingredients with outstanding advantages in regulating body functions and exhibit diverse antihypertensive effects. Thus, Chinese herbal medicines are expected to overcome the limitations of traditional medicines with a single active ingredient [24]. TJT (No. Z20110021) is a Chinese medicine patented and registered by the Shandong Food and Drug Administration, and it has been well recognized and used in the National TCM Clinical Research Base and affiliated hospitals for hypertension patients.

TJT is obtained by extracting the effective parts of *Uncaria rhynchophylla* and *Semen Raphani*, two Chinese medicines [17]. Initially, we systematically investigated [25–28] TJT’s principal pharmacological components, *Uncaria rhynchophylla* total alkaloids (including rhynchophylline and isorhynchophylline), and *Semen Raphani* soluble alkaloids (mainly sinapine thiocyanate) and discovered that their principal mechanisms of activity are as follows. Lower plasma ET 1 levels increase plasma NO levels, adjust the balance of NO/ET, reduce plasma renin and angiotensin II levels, and negatively regulate the activity of the RAAS system in rats. Furthermore, the expression levels of serum small-molecule metabolites and proteins [15–18] confirmed that TJT has potential antihypertensive effects and diverse cardiovascular protective effects, including reducing vascular endothelial cell inflammation and antioxidation and improving endothelial function. In addition, in our multicenter clinical trial of TJT for the treatment of stage 1 hypertension with hyperactivity of liver-yang syndrome, we found that TJT has a clear antihypertensive effect on patients with stage 1 hypertension. These findings and observations provide momentum for a large-sample controlled trial of TJT-assisted Val/Aml SPC for stage 2 hypertension to evaluate the efficacy and safety of TJT.

## Strengths and limitations

The efficacy and safety of the Val/Aml combination have been demonstrated in large randomized controlled trials [29, 30]. To the best of our knowledge, there is no treatment based on Val/Aml as a proprietary Chinese medicine for patients with grade 2 hypertension involving a multicenter, blinded, randomized, placebo-controlled clinical trial to evaluate the proprietary Chinese medicine TJT as an aid in the use of Val/Aml, whether in the treatment of 4 weeks to promote the early attainment rate of BP in stage 2 hypertension patients and improving the clinical symptoms of the patients. We hope that this trial will provide high-quality evidence for the efficacy and safety of TJT in the treatment of grade 2 hypertension. The limitation of this study is that it will be carried out in only Shandong Province, China, and the research period is relatively short. Therefore, we have no interim analyses or stopping guidelines. The results of this study still need to be confirmed by clinical research practice.

## Trial status

This protocol (version 2.0 date 10 January 2020) was approved by the clinical research ethics committee of Xue et al. The study started on 8 July 2020; thus far, 57 patients have been recruited. The study will be finished by December 2021.

## Supplementary Information

**Additional file 1.** SPIRIT checklist.

**Additional file 2.** Research settings and name of each ethics committee.

**Additional file 3.** TCM Syndrome Integral Scale.

**Additional file 4.** Informed consent form.

## Abbreviations

TJT: Tengfu Jiangya tablet
Val/Aml: Valsartan/amlodipine
TCM: Traditional Chinese medicine
RCT: Randomized controlled trial
CRF: Case report form
GCP: Good Clinical Practice
AE: Adverse event
RCT: Randomized clinical trial
MSSBP: Mean sitting systolic blood pressure
MSDBP: Mean sitting diastolic pressure
SFDA: State Food and Drug Administration
TSC: Trial steering committee
