# Supplementary material for: Efficacy and safety of Tengfu Jiangya tablet combined with valsartan/amlodipine in the treatment of stage 2 hypertension: study protocol for a randomized controlled trial
Source: Trials. 2022 Feb 22;23:171. doi: 10.1186/s13063-022-06089-z (PMC8864829; doi:10.1186/s13063-022-06089-z)
Supplement: Supplementary file 2 — Additional file 2. Research settings and name of each ethics committee. [file 13063_2022_6089_MOESM2_ESM.docx]

Table 1 Research settings and name of each ethics committee

| Research setting | Ethics committee name | Approval registration  number |
| --- | --- | --- |
| Affiliated Hospital of Shandong University of Traditional Chinese Medicine | Ethics Committee of Affiliated Hospital of Shandong University of Traditional Chinese Medicine | （2019）伦审第（064）号-KY |
| The Third Affiliated Hospital of Shandong First Medical University | Ethics Committee of the Third Affiliated Hospital of Shandong First Medical University | FY2020021 |
| Qingdao Hospital of Traditional Chinese Medicine | Ethics Committee of Qingdao Traditional Chinese Medicine Hospital | （2020）伦审【科第】（032）号 |
| Zibo city central hospital | Ethics Committee of Zibo Central Hospital | 20200800 |
| Yantai Hospital of Traditional Chinese Medicine | Ethics Committee of Yantai Traditional Chinese Medicine Hospital | （2020）伦审（004）号-KY |
| Penglai Hospital of Traditional Chinese Medicine | Ethics Committee of Penglai Traditional Chinese Medicine Hospital | （2020）伦审（037）号-KY |
| Yinan Hospital of Traditional Chinese Medicine | Ethics Committee of Yinan Hospital of Traditional Chinese Medicine | LLPJ2020-1 |
