# Supplementary material for: Efficacy and safety of Tengfu Jiangya tablet combined with valsartan/amlodipine in the treatment of stage 2 hypertension: study protocol for a randomized controlled trial
Source: Trials. 2022 Feb 22;23:171. doi: 10.1186/s13063-022-06089-z (PMC8864829; doi:10.1186/s13063-022-06089-z)
Supplement: Supplementary file 3 — Additional file 3. TCM Syndrome Integral Scale. [file 13063_2022_6089_MOESM3_ESM.pdf]

高血压病肝阳上亢证诊断量表

为更加细致了解患者病情，以下问题是关于近两周来患者的一些主观感受。请调查人员详细询问患者感受后如实填写，在合适的选项前划“√”，条目 1~11 中①~⑤分别计以 1~5 分，条目 12~18 中①计 1 分，②计 3 分。

量表总分计算公式：Y=11 头晕+12 头痛+1 耳鸣+10 口苦+6 口干+2 胁肋疼痛+2 多梦+2 失眠+13 急躁易怒+4 大便秘结+6 小便黄+5 面红+4 目赤+5 舌质红+5 舌苔黄+6 脉弦+4 脉数+3 脉有力；当高血压病患者的证候积分≥230 时，可诊断为肝阳上亢证。谢谢！

1.您有头晕的感觉吗？

①根本没有　②有，较轻　③有，一般　④比较严重　⑤很严重

2.您有头痛的感觉吗？

①根本没有　②有，较轻　③有，一般　④比较严重　⑤很严重

3. 您有耳朵里鸣响的感觉吗？

①根本没有　②有，较轻　③有，一般　④比较严重　⑤很严重

4. 您觉得口中发苦吗?

①根本没有　②有，较轻　③有，一般　④比较严重　⑤很严重

5. 您有口干的感觉吗？

①根本没有　②有，较轻　③有，一般　④比较严重　⑤很严重

6. 您肋骨部位最近有疼痛的感觉吗？

①根本没有　②有，较轻　③有，一般　④比较严重　⑤很严重

7. 您最近睡觉的时候做梦多吗？

①根本没有　②有，较轻　③有，一般　④比较严重　⑤很严重

8. 您最近失眠吗？

①根本没有　②有，较轻　③有，一般　④比较严重　⑤很严重

9. 您最近容易急躁发怒吗？

①根本没有　②有，较轻　③有，一般　④比较严重　⑤很严重

10. 您最近大便干吗？

①根本没有　②有，较轻　③有，一般　④比较严重　⑤很严重

11. 您最近小便发黄吗？

①根本没有　②有，较轻　③有，一般　④比较严重　⑤很严重

以下内容由医生填写：(请划“√”)

12. 面色　红　①无　②有

13. 眼睛发红　①无　②有

14. 脉弦　①无　②有

15. 脉有力　①无　②有

16. 脉数　①无　②有

17. 舌质　红　①无　②有

18. 舌苔　黄　①无　②有

量表总分=_________________

辨证结果：　肝阳上亢证：　☐是　☐否

观察医师签名：_______________　　日期：20______年____月____
