# Supplementary material for: Efficacy and safety of Tengfu Jiangya tablet combined with valsartan/amlodipine in the treatment of stage 2 hypertension: study protocol for a randomized controlled trial
Source: Trials. 2022 Feb 22;23:171. doi: 10.1186/s13063-022-06089-z (PMC8864829; doi:10.1186/s13063-022-06089-z)
Supplement: Supplementary file 4 — Additional file 4. Informed consent form. [file 13063_2022_6089_MOESM4_ESM.docx]

知情同意书·知情告知页

尊敬的受试者：

您的医生已经确诊您患有2级原发性高血压。我们邀请您参加山东中医药大学附属医院临床研究基地建设项目子课题：“藤菔降压片联合缬沙坦/氨氯地平治疗原发性高血压（2级）肝阳上亢证的随机、双盲、安慰剂对照、多中心临床研究”。本研究已经得到山东中医药大学附属医院伦理委员会的审查和批准。

研究介绍

一、研究背景和研究目的

原发性高血压是最常见的心血管疾病，据2018年中国高血压调查最新数据显示患病率总体呈增高的趋势。高血压内在发病机制复杂，临床上患者常需联合应用多种降压药物，长期使用多种药物后的不良反应及经济负担难以避免。

祖国医学将“高血压”归属于 “眩晕”、“头痛”范畴，肝阳上亢证是最常见的证型，表现为眩晕、头痛等证候。“藤菔降压方” 为高血压国家中医临床研究基地治疗该病证的有效中药复方制剂，由钩藤和莱菔子两味中药组成。为提高治疗效果，在中医药理论的指导下，优选剂量配比，采用现代制剂工艺提取凉藤菔降压方中抗高血压有效组分，研制成院内制剂—藤菔降压片（鲁药制字Z20110021），经药理学实验证实该药在药效剂量范围内，临床用药安全有效。现为进一步明确藤菔降压片的临床疗效及安全性，按照《药物临床试验设计规范》进行本课题设计，以指导临床治疗。

本研究采用多中心、随机、双盲、安慰剂平行对照的临床研究方法，旨在评价藤菔降压片联合缬沙坦/氨氯地平干预原发性高血压（2级）肝阳上亢证临床疗效和安全性，为临床治疗提供客观证据。

本研究将在山东中医药大学附属医院、烟台市中医医院、青岛市中医院、蓬莱市中医院等研究中心进行，预计有288名受试者自愿参加。

二、哪些人适宜参加研究

1. 年龄在18-65岁之间；

2. 同时符合高血压病西医诊断标准和肝阳上亢证中医证候辨证标准；

3. 经高血压病的血压水平、心血管风险分层确定为2级原发性高血压、低危和中危患者，血压符合以下3条中的任意1条：

（1）收缩压160-179mmHg和(或)舒张压100-109 mmHg，既往未服用任何降压药物，未见心脑肾等并发症；

（2）已明确诊断为原发性高血压，经改善生活方式或者其它形式的非药物治疗3个月血压仍未达标，维持在160-179/100-109mmHg者；

（3）已明确诊断为原发性高血压，经改善生活方式或者其它形式的非药物治疗并口服降压药物治疗3个月血压仍未达标，维持在160-179/100-109mmHg者；

4.患者明确诊断为高血压病的病程必须大于3个月；

5.知情同意并签署知情同意书者。

三、哪些人不宜参加研究

1.各种继发性高血压病；

2.近3个月内曾接受其它新药临床实验者；

3.中重度糖尿病患者或半年内有心肌梗死或有脑卒中史者；

4.妊娠或准备妊娠者以及哺乳期妇女；

5.既往对多种药物过敏者或者过敏体质者；

6.合并有精神病、酗酒和或精神活性物质药物滥用者和依赖者；

7.同时合并以下器官损害或疾病者：心绞痛、心力衰竭、一过性脑供血不足、高血压性脑病、视网膜病变(伴或不伴有视乳头水肿)、血浆肌酐浓度2.0mg/dl 以上肾功衰竭、肝功能衰竭、主动脉夹层或主动脉瘤，动脉闭塞性疾病症状明显者。

注：以上各项满足任何1项或多项均应排除，不能入选。

四、研究过程

如果您愿意参加本项研究，您将有可能接受以下2种治疗方案：研究组治疗：藤菔降压片联合缬沙坦/氨氯地平药物治疗；对照组治疗：藤菔降压片安慰剂联合缬沙坦/氨氯地平治疗。我们会在您接受治疗的4周内定期对您进行血压、心率、心电图检查，血、尿、便常规检查（动态血压检测主要在山东中医药大学附属医院分中心进行），肝肾功能等安全指标检查，血脂（TG、LDL）、血糖、尿微量白蛋白、尿酸、超敏C反应蛋白等疗效指标；并会在治疗结束后8周内对您进行随访。

五、您的权利和利益

（1）是否参加研究完全取决于您的意愿。您可以拒绝参加此项研究，或在研究过程中的任何时间退出本研究，这都不会影响您和医生间的关系，都不会影响对您的医疗或有其他方面利益的损失。 您的医生或研究者出于对您的最大利益考虑，可能会随时中止您参加本项研究。您可以不参加本项研究，或中途选择退出研究。如果您决定退出本研究，请与您的医生联系，您可能被要求进行相关检查，这对保护您的健康是有利的。

（2）参加本项研究，您的病情有可能获得改善，本项研究还有助于确定哪种治疗方法可以更安全有效地治疗与您患有相似病情的其他病人。

（3）您可免费接受血压、心率、心电图、血、尿、肝肾功能等与实验相关的检查；您可免费接受中药藤菔降压片及缬沙坦/氨氯地平治疗；您可免费获得高血压病相关的医学指导。

（4）已经有证据提示：缬沙坦/氨氯地平片对2级高血压患者的的血压控制率，耐受性良好。藤菔降压片治疗原发性高血压同时符合高血压病西医诊断标准和肝阳上亢证中医证候辨证标准证有满意的疗效，但这并不能保证本研究方案对您肯定有效。本研究所采用的藤菔降压片也不是治疗原发性高血压的唯一的方法。如治疗方案对您的病情无效，您可以向医生询问有可能获得的替代治疗方法。我们将通过定期检查藤菔降压片治疗可能引起的副作用/不良反应，医生将尽全力预防和治疗由于本研究可能带来的伤害。

（5）如果在临床试验中出现与本实验相关的不良事件。课题组将按照我国《药物临床试验质量管理规范》的规定对与试验相关的损害提供治疗的费用及相应的经济补偿。对于您同时合并的其他疾病所需的治疗和检查，将不在免费的范围之内。如果因观察药物对降低血压及改善症状无效，我们将提供其他药物一个疗程的免费治疗。

六、参加研究有哪些风险

研究表明，藤菔降压片治疗高血压安全性良好，未见明确不良反应，但由于医学科学的复杂性和患者个体之间的差异性，仍不排除有未知的不良反应。

如果在研究期间您出现任何不适，或病情发生新的变化，或任何意外情况，不管是否与研究有关，均应及时通知您的医生，他/她将对此作出判断并给与适当的医疗处理。此外，服用藤菔降压片可能出现无效的情况，以及因治疗无效或者因合并其他疾病等原因而导致病情继续发展。在研究期间，如果医生发现本项研究所采取的藤菔降压片对降低血压及改善症状无效，将会中止研究，改用其他可能有效的治疗措施。

七、保密性

您的医疗记录（研究病历/CRF、化验单等）将完整地保存在山东中医药大学附属医院。医生会将化验检查结果记录在您的门诊病历上。研究者、申办者代表、伦理委员会和药品监督管理部门将被允许查阅您的医疗记录。任何有关本项研究结果的公开报告将不会披露您的个人身份。我们将在法律允许的范围内，尽一切努力保护您个人医疗资料的隐私。

除本研究以外，有可能在今后的其他研究中会再次利用您的医疗记录。您现在也可以声明拒绝除本研究外的其他研究利用您的医疗记录。

八、受试者须知

是否参加本项研究由您自己决定。您可以和您家人或者朋友讨论后做出决定。在您做出参加研究决定前，请尽可能向您的医生询问有关问题，直至您对本项研究完全理解。感谢您阅读以上材料。若您仔细阅读上述内容后仍存在疑义或拒绝参加，则将不被纳入课题组。如果您决定参加本项研究，请告诉您的医生，他/她会为您安排一切有关研究的事务。请您保留这份资料。

Informed Consent Page

Dear subjects:

Doctor has confirmed that you have grade 2 essential hypertension. We invite you to participate in the sub-project of the clinical research base construction project of the affiliated Hospital of Shandong University of traditional Chinese Medicine: "Randomized, double-blind, placebo-controlled, multicenter clinical trial of Tengfu Jiangya tablet combined with valsartan / amlodipine in the treatment of essential hypertension (grade 2) with hyperactivity of liver-yang." This study has been reviewed and approved by the Ethics Committee of the affiliated Hospital of Shandong University of traditional Chinese Medicine.

1. Background and objective

Essential hypertension is the most common cardiovascular disease. According to the latest data of 2018 Chinese hypertension survey, the overall prevalence rate shows an increasing trend. The inherent pathogenesis of hypertension is complex, clinical patients often need to combine the use of a variety of antihypertensive drugs, long-term use of a variety of drugs after adverse reactions and economic burden can not be avoided.

In traditional Chinese medicine, "hypertension" belongs to the category of "vertigo" and "headache". The syndrome of hyperactivity of liver-yang is the most common syndrome, such as vertigo, headache and so on. " Tengfu Jiangya tablet " is an effective compound preparation of traditional Chinese medicine for the treatment of this syndrome in the national clinical research base of traditional Chinese medicine for hypertension, which is composed of Uncaria and semen Raphani. In order to improve the therapeutic effect, under the guidance of the theory of traditional Chinese medicine, the dosage ratio was optimized, and the antihypertensive effective components of Tengfu Jiangya tablet prescription were extracted by modern preparation technology, and the hospital preparation- Tengfu Jiangya tablet (Z20110021) was developed. it is proved by pharmacological experiments that the drug is safe and effective in clinical use within the range of efficacy and dose. In order to further clarify the clinical efficacy and safety of Tengfu Jiangya tablet, this subject is designed according to the "Drug Clinical trial Design Code" to guide clinical treatment.

A multicenter, randomized, double-blind, placebo-controlled clinical study was conducted to evaluate the clinical efficacy and safety of Tengfu Jiangya tablet combined with valsartan / amlodipine in the treatment of essential hypertension (grade 2) with hyperactivity of liver-yang. To provide objective evidence for clinical treatment.

This study will be conducted in the affiliated Hospital of Shandong University of traditional Chinese Medicine, Yantai Hospital of traditional Chinese Medicine, Qingdao Hospital of traditional Chinese Medicine, Penglai Hospital of traditional Chinese Medicine and other research centers. 288 subjects are expected to participate voluntarily. 2. Inclusion criteria

（1）The age is between 18 and 65 years old;

（2）At the same time, it conforms to the diagnostic standard of western medicine for hypertension and the standard of TCM syndrome differentiation for hyperactivity of liver-yang.

（3）According to the stratification of blood pressure level and cardiovascular risk of hypertension, the patients with grade 2 essential hypertension, low-risk and moderate-risk patients' blood pressure conformed to any one of the following three items:

① Systolic blood pressure 160mm-179mmHg and / or diastolic blood pressure 100-109mmHg. They had not taken any antihypertensive drugs in the past and had no complications such as heart, brain and kidney.

② Those who had been clearly diagnosed as essential hypertension and whose blood pressure had not reached the standard after lifestyle improvement or other forms of non-drug treatment for 3 months, maintained at 160-179 109mmHg;

③ Those who had been clearly diagnosed as essential hypertension and had not reached the standard after lifestyle improvement or other forms of non-drug treatment and oral antihypertensive drug treatment for 3 months, maintained at 160-179 ppm 100-109mmHg;

(4) those who had been clearly diagnosed as essential hypertension and had not reached the standard after 3 months of lifestyle improvement or other forms of non-drug treatment and oral antihypertensive drug treatment. The course of a patient clearly diagnosed as hypertension must be more than 3 months;

5. Those who give informed consent and sign the informed consent form.

3. Exclusion criteria

（1）Various secondary hypertension;

（2）Those who have received clinical trials of other new drugs in the past 3 months;

（3）Patients with moderate and severe diabetes or those with a history of myocardial infarction or stroke within half a year;

（4）Pregnant or preparing for pregnancy and lactating women;

（5）People who are allergic to a variety of drugs or who are allergic to various drugs in the past;

（6）Combined with psychotic, alcoholic and or psychoactive substance abusers and addicts;

（7）At the same time, patients with the following organ damage or diseases: angina pectoris, heart failure, transient cerebral insufficiency, hypertensive encephalopathy, retinopathy (with or without optic papilla edema), plasma creatinine concentration above 2.0mg/dl renal failure, liver failure, aortic dissection or aortic aneurysm, obvious symptoms of arterial occlusive disease. Note: any one or more of the above items should be excluded and cannot be selected.

4. The course of the study

If you are willing to participate in this study, you are likely to receive the following two treatment options: the study group is treated with Fujimi antihypertensive tablets combined with valsartan / amlodipine; the control group was treated with Fujimi antihypertensive tablets combined with valsartan / amlodipine. We will check your blood pressure, heart rate, ECG, blood, urine and stool regularly within 4 weeks of your treatment (ambulatory blood pressure test is mainly carried out in the sub-center of the affiliated Hospital of Shandong University of traditional Chinese Medicine), liver and kidney function, blood lipid (TG, LDL), blood glucose, urinary Microalbumin, uric acid, hypersensitive C-reactive protein and other efficacy indicators; and you will be followed up within 8 weeks after the end of treatment.

5. The rights and interests

(1)Whether or not to participate in the research depends entirely on your wishes. You may refuse to participate in this study, or withdraw from this study at any time in the course of the study, which will not affect your relationship with your doctor, nor will it affect the loss of your medical or other benefits. Your doctor or researcher may suspend your participation in this study at any time in your best interest. You can opt out of this study or opt out of the study halfway. If you decide to withdraw from this study, please contact your doctor and you may be asked to have a relevant examination, which is beneficial to protect your health.

(2) By participating in this study, your condition may be improved, and this study will also help to determine which treatment is more safe and effective to treat other patients with similar conditions.

(3) You can receive free tests related to the experiment, such as blood pressure, heart rate, ECG, blood, urine, liver and kidney function, etc., and you can receive free treatment with traditional Chinese medicine tendril antihypertensive tablets and valsartan / amlodipine. You can get medical guidance related to hypertension free of charge.

(4) There has been evidence that valsartan / amlodipine tablets are well tolerated in patients with grade 2 hypertension. Tengfu Jiangya tablet in the treatment of essential hypertension at the same time in line with the standard of western medicine diagnosis of hypertension and the standard of TCM syndrome differentiation of liver-yang hyperactivity syndrome has a satisfactory effect, but this does not guarantee that this research scheme is definitely effective for you. The Fujita antihypertensive tablets used in this study are not the primary treatment.

(5) If there are adverse events related to this experiment in the clinical trial. The research group will provide treatment costs and corresponding economic compensation for the damage related to the trial in accordance with the provisions of the quality Management Standard of Drug Clinical Trials in China. The treatment and examination required for other diseases that you combine at the same time will not be free of charge. If the observation drug is not effective in lowering blood pressure and improving symptoms, we will provide a free course of treatment for other drugs.

6. What are the risks of participating in the study?

Studies show that Tengfu Jiangya tablet are safe in the treatment of hypertension, and there are no clear adverse reactions, but unknown adverse reactions are still not ruled out because of the complexity of medical science and the differences between individual patients. If you have any discomfort, new changes in your condition, or any unexpected circumstances during the study, whether related to the study or not, you should inform your doctor in a timely manner, and he / she will make a judgment and give appropriate medical treatment. In addition, taking Fujita antihypertensive tablets may be ineffective, and the disease may continue to develop due to ineffective treatment or other diseases. During the study, if doctors find that Fujita antihypertensive tablets taken in this study are ineffective in lowering blood pressure and improving symptoms, they will discontinue the study and switch to other treatment measures that may be effective.

7. Confidentiality

Your medical records (research medical records / CRF, laboratory sheets, etc.) will be kept intact in the affiliated Hospital of Shandong University of traditional Chinese Medicine. The doctor will record the test results on your outpatient record. Researchers, sponsor representatives, ethics committees and drug regulatory authorities will be allowed to access your medical records. Any public reports on the results of this study will not disclose your personal identity. We will make every effort to protect the privacy of your personal medical data within the limits permitted by law. In addition to this study, it is possible that your medical records will be used again in other future studies. You can now also declare that you refuse to use your medical records for studies other than this study.

8. Subjects should know

It is up to you to decide whether to participate in this study or not. You can discuss it with your family or friends and make a decision. Before you decide to participate in the study, please ask your doctor as much as possible until you fully understand the study. Thank you for reading the above materials. If you still have doubts or refuse to participate after reading the above carefully, you will not be included in the research group. If you decide to participate in this study, please tell your doctor that he or she will arrange everything related to the research for you. Please keep this information.
